# Colds—Their Causes and Treatment1Read at the forty-third annual meeting of the Central New York Medical Association held at Syracuse, October 19 and 20, 1910.

**Published:** 1911-08

**Authors:** Sargent F. Snow

**Affiliations:** Syracuse, N. Y.


### Colds—Their Causes and Treatment[1]

By SARGENT F. SNOW,
Syracuse, N. Y.

EVERY otologist and laryngologist who attempts to handle a chronic case has before him at once the problem of controlling the many acute colds of inflammations that occur in the membranes of the nose and throat.

These inflammatory actions are often so slight that the patient has not noticed them, but we find them so frequently that they interfere seriously with our efforts to the extent that often in ear affections it is unwise to encourage local treatment.

For fifteen years the reader has groped after the cause of these recurring membrane disturbances thinking at first they were caused entirely by morbid intra-nasal states, but later decided that there was always an underlying systemic factor to be held responsible. What exact trouble this was seemed obscure, and it was only since I began a closer study of Auto-Intoxication four years ago, that I have felt I had a sufficient grasp of the situation to say what this systemic factor was.

In opening the subject of head colds I must ask your indulgence for not quoting authorities and literature, as I have been unable to find time to take up the matter from that standpoint. This attempt then can in no way be called classical, but I earnestly ask that the views set forth shall have a few moments of your thoughts.

Auto-toxic colds, colds from self-poisoning, or common colds if you like, in the opinion of the reader is a condition induced primarily by toxic substances developed within our own body, a self-poisoning from our own bacteria, or in other words, a nitrogenous auto-intoxication of microbic origin.

---

1 Read at the forty-third annual meeting of the Central New York Medical Association held at Syracuse, October 19 and 20, 1910.

Active inflammation and fever usually mark the limit of toleration by the system for toxic materials. Such an inflammation is an auto-toxic explosion or crisis of elimination; in other words, a *cold* is the expression of an *auto-intoxication* showing itself in the mucous membranes. Those infective inflammations or influenzas produced by bacilli developed outside of of the body, as pointed out by Combe some years ago, are more properly termed Intoxinations, not Intoxications.

It is now a well accepted fact that our intestinal tract is supplied with digestive bacilli that are capable of becoming very abundant and pernicious if not kept under proper subjection by active secretions from the intestinal mucosa, liver and anti-toxic glands. These three barriers protect the body from the harmful actions of those digestive bacteria while our glands are kept in healthy activity, but if the healthy activity of the intestinal mucosa and liver is impaired, then our body is not producing anti-toxins sufficient to protect us and we become a prey to our own bacteria. The system then becomes Auto-Toxic.

According to Combe on Auto-Intoxication, (see page 9), Marfan is quoted as follows:—"We may say that the microbes intervene actively in all digestive processes, but that besides their undeniably useful role, it is also undeniable that their action transforms the digestive canal, even in the normal state, into a receptacle and constant laboratory of poisons."

It is small wonder then if they overwhelm us when our body functions are torpid or depressed.

Some people appear to possess a remarkable tolerance for toxic accumulation before it is shown in an irritable or inflamed state of the head membranes, while others quickly show such evidences.

In the first mentioned we find them so loaded with toxic materials that we must at once get their emunctories into vigorous action if we save them from a severe sickness, others will respond to a simple clearing out of the intestinal canal.

In active head colds or pharyngeal inflammation we are likely to be forced to unload the system rapidly, not only by the bowels, but by the skin and kidneys. Acid intoxications can be materially reduced by alkaline eliminative solvents. Hot baths and the usual remedies for inducing free perspiration can be made of great help. The whole question becomes one of rapid elimination.

As a rule patients that are auto-toxic frequently take colds and have a holy fear of draughts; observation will soon show that their tongue is coated, breath likely to be foul, whites of eyes dull or yellow, complexion dirty grey and lips too red, or they will show a history of at least irregular bowel action and more often give a history of chronic constipation.

We need have no fear of draughts or exposure except when we are loaded with enterotoxins or are greatly fatigued. The draught fetish is a constant menace to our well being. It is true that a person saturated with toxic materials is very sensitive to to cold draughts and should be protected, but only until the toxic materials can be unloaded, then give him fresh air in wholesale quantities.

While we may have sneezing and a nose stuffed from sitting in a draught, the congestion induced will pass off readily upon moving about, unless the nasal passages are abnormal or the system is toxaemic.

If it should occur that our patient has a nose obstructed and made unduly sensitive by a deflected septum, outgrowth, or turbinal enlargements, the only reasonable procedure is to have these abnormalties corrected. Such intra-nasal defects do not cause actual colds, but they induce such an irritable and occluded state of the respiratory passages, that a very slight systemic disturbance will produce marked and continued symptoms.

Under present modes of diet and life, it is a problem indeed to eat and live so that we do not develop a nitrogenous auto-intoxication. It would seem that the human race either had inherited defective digestive functions, or that our food was far from being the proper sort. Either we must correct the diet or we must anticipate auto-toxic accumulations by administering a glandular stimulating remedy at such intervals as will prevent an auto-toxic explosion or crisis. The author finds it necessary to take such a remedy every ten days or two weeks, even though he lives plainly and has regular movements of the bowels twice a day. Many patients will find it necessary to anticipate such a loading up of toxins by an active stimulant to the liver and upper bowel secretions once a week, some once in two weeks. Such a course gives them protection from *intoxication* through poisons developed *outside* the body, and from *intoxications* through poisons developed *inside* the body. It also soon brings them into a state where a fear of draughts and exposure has become a thing of the past.—Fresh air has become a luxury instead of a menace. —An unwholesome individual has become a being of health and pleasure, not one who insists that every window shall be closed, and his or her associates subjected to the discomforts of a room over-heated and poorly ventilated.

It is repeatedly proven to us that our body in a healthy state is an efficient anti-toxic laboratory. Note the extensive operative work that can be done on the mucous membranes of the head without subsequent antiseptic dressings being necessary, simply have our instruments clean and the mucous secretions will take proper care of the wound under all ordinary conditions.

Whether an acute rhinitis or head cold comes from a poison developed inside the body, or comes from a poison developed outside the body, like the influenzas, La Grippe, and the like, the treatment can well be along the same lines, the objective point is the same, to subdue pernicious bacteria by augmenting anti-toxic secretions.

A cold is really one of nature's kindliest warnings that our body is becoming surcharged with poisons. The head membranes are good barometers of the system, and prompt heed given to their warnings will greatly reduce our death rate and suffering from rheumatism, tuberculosis, appendicitis, acute indigestion, renal and other functional disorders.

If we, as a profession, had already mastered the problems of correct living there would be little need of medicine. If we knew how to select, prepare and apportion our foods but few diseases would bother us, as diseases are largely preventable. We have not solved these problems and will not for many years. The culinary art aims more to tickle the palate than to furnish nourishment, and until greater improvement is wrought along these lines, Materia Medica will have to be a succoring angel.

Acepting, then, the fact that to prevent or cure colds we must fight digestive irregularities and resulting toxic activities, we approach these lines of combat in a rational and scientific way.

If our patient is eating too largely of meat and heavy nitrogenous foods, such a diet must be corrected by substituting the proper proportions of lighter starchy foods; if he is overeating, or taking too much fluid with his meals, those things should be looked into, for intestinal putrefaction is frequently caused by such erroneous habits.

Eating too largely of starchy foods causes fermentation in the small intestines; too largely of albuminoids brings putrefaction in the large intestines; each capable of exciting strong systemic disturbances to the end that we must either solve our patient's problems by prevention through correction of diet, or by giving him relief medicinally, and we all know how unsatisfactory medical relief is except as a temporary expedient.

Preventive knowledge is the great goal of our achievement—our ideal course of study—but we must be practical and until we possess that preventive knowledge more completely and can indicate more definitely the correct dietary modes of life, we must rely upon such relief as can be obtained through medicines.

If we are to combat toxic activities we must be armed with anti-toxins, and as our body anti-toxins are produced by our glands and mucosa, we must administer glandular stimulants to make our body manufacture more abundant and active anti-toxic secretions. It is not necessary for me to say what glandular stimulant shall be used, calomel seems to be most efficient, though I use it only in extreme or obstinate cases, preferring as a rule a blue mass and colocynth compound. Whatever is chosen we should be sure is followed by a prompt and copious clearing out of the bowels, and the dose repeated every two or three days until the tongue is clean, the breath sweetened and the complexion less muddy. Recurrences should be prevented by anticipating auto-toxic crises as before mentioned.

It is the author's practise to give a medium dose of castor oil or salines between the doses of glandular stimulant, if the auto-intoxication is persistent or active and calomel has been used, the aim being of course to prevent cumulative effects from the mercurial. Under such a plan of medical treatment I have found no untoward results, on the contrary, am each year more and more satisfied, and often surprised, with the general good such treatment does the patient, besides preventing subsequent membrane congestion.

If we are auto-toxic we take cold easily, if we are not auto-toxic we do not take cold; exceptions to this rule are few, Auto-Toxic colds are common.

I regret exceedingly that there are not more digestive specialists throughout the country to relieve us of these auto-intoxicated patients, but until there are we will have to make the best of the situation. Such cases, with their complex problems, are really subjects for more careful painstaking observations than can be given by a busy operative otologist or laryngologist, particularly so if he attempts to go into the details of diet correction. It is quite easy though to note how often the tongue of our throat or ear patient coats up, complexion becomes muddy, appearances indicate billiousness, and the head membranes take on increased congestion, sure indications that toxins are again accumulating in the system and that we should anticipate the impending crisis by prompt acting glandular stimulant. If such toxic accumulations occur every two weeks, we can wake up the anti-toxic functions a few days earlier, if every ten days, we can give our anti-toxic stimulant once a week, always adapting the medicine to the case.

A glandular stimulant can be given for years with no harmful result if proper combination is used and reasonable care taken that the patient does not take the remedy as a daily cathartic.

The author has used a pill composed of blue mass, colocynth compound, strychnia sulphate and belladonna for ten years, with not one instance of mecurial poisoning, nor one instance tending to show that a constipated habit has been induced. Such a drug combination does not require an after flushing with salines, and as stated earlier in the paper, is sufficiently effective except in extreme or obstinate cases where nothing acts so well as calomel. When a chronic constipation is complained of, some laxative like the Aloin, Strychnia and Belladonna pill can be used each night with propriety.

If we are able to handle and prevent acute inflammation of the respiratory membranes known as common colds by recognising and treating the patients' auto-intoxication, what may we not hope will sometime be done with the more grave affections? It appears to the author like the dawn of a new era and he hardly dares let his fancy travel.

It is possible yet that the medical practitioner may outstrip his co-laborer, the laboratory scientist, in the race to save life and promote human longevity. Both are engaged in legitimate effort, the one to produce anti-toxins or antigens outside the body for injection in emergencies, the other to induce anti-toxic secretions within the body for immunity and cure.

